# A proposed framework to limit post-lockdown community transmission of COVID-19 in Africa

**DOI:** 10.11604/pamj.2021.38.303.24008

**Published:** 2021-03-23

**Authors:** Eric Nzirakaindi Ikoona, David Lagoro Kitara

**Affiliations:** 1 ICAP at Columbia University, Freetown, Sierra Leone,; 2 Gulu University, Faculty of Medicine, Department of Surgery, Gulu, Uganda,; 3 Harvard T.H. Chan School of Public health, Department of Global Health, Boston, USA

**Keywords:** COVID-19, SARS-CoV-2, Africa, lockdown, easing, restrictions, pandemic

## Abstract

As of March 11, 2021, 3,992,044 coronavirus disease 2019 (COVID-19) cases and 106,615 deaths (case fatality rate 2.67%) have been reported on the African continent. In March 2020, even before the first case of COVID-19 was registered, some African countries implemented total lockdown measures, which limited movement of people, banned mass gatherings, and closed schools and borders. However, these control measures, which affect individuals and society's well-being, cannot be implemented for a long time. There is an urgent need for a robust framework to guide African countries to make evidence-based decisions on easing these restrictive measures and reapply them when the infection rates increase significantly. This article presents a proposed framework to guide lockdown easing while limiting the community spread of COVID-19 in Africa. Due to lack of information on the impact of relaxing restrictions on peoples' movement on the spread of severe acute respiratory syndrome coronavirus 2 (SARS-CoV-2, the causative agent for COVID-19) and how businesses will respond, it is almost clear that there is no single grand lockdown exit strategy. African governments should develop flexible, iterative lockdown exit plans based on epidemiological disease data, economic indicators, and peoples' views to inform decisions, all of which are paramount for success. A phased approach of changes and willingness to adapt methods will allow governments to minimize the pandemic's adverse impact and respond accordingly as new control tools become available.

## Commentary

As of March 11, 2021, a total of 3,992,044 coronavirus disease 2019 (COVID-19) cases and 106,615 deaths (case fatality rate 2.67%) have been reported on the African continent. These include 2,891,840 cases and 73,943 deaths in the World Health Organization (WHO) African Region and 110,204 cases and 32,672 deaths in the WHO Eastern Mediterranean Region [1]. The African continent is sub-divided into two WHO Regions: The WHO African Region (consisting of 47 of the 54 African member states) and WHO Eastern Mediterranean Region (consisting of 7 African countries; Djibouti, Egypt, Libya, Morocco, Somalia, Sudan, and Tunisia) [1]. The WHO African Region had 2.45% (2,891,840/117,799,587) of the worldwide COVID-19 cases, even though it represents 13.7% of the global population, and 2.83% (73,943/2,615,018) of deaths, with a case fatality rate of 2.56% (73,943/2,891,840), compared with 2.39%(826,911/34,664,874) in the European Region and 2.22% (2,615,018/117,799,584) globally [1].

Surveillance data from WHO indicates that most African countries already have local transmission of COVID-19, and the number of countries with widespread community transmission is also increasing [1]. As early as March 2020, governments across Africa, for example, Rwanda, Kenya, South Africa, Botswana, and Uganda, had already instituted multiple public health measures. The control measures instituted included enhanced surveillance to detect cases, contact tracing, quarantine, isolation, stakeholder engagement, sensitization of the public, and case management to limit the spread and adverse outcomes of COVID-19 among citizens [2,3]. Also, 77.8% (42/54) of the African continent countries have implemented stringent physical and social distancing measures, including movement restrictions to limit contacts among people [2]. These types of restrictions are often referred to as “lockdowns” and are meant to slow COVID-19 transmission [2]. Lockdown measures instituted in African countries (for example, in Uganda, Rwanda, and South Africa) have included quarantines, stay home policy, closure of borders (airports, land, and water crossing points), and closure of congregate settings such as places of worship, schools, markets, salons, public transport, night curfews, and sports stadia, among others [3]. Through forced physical distancing, lockdown measures have in most occasions successfully curbed the spread of COVID-19 within communities [2,3].

However, these measures have also inadvertently hurt the social and economic life of individuals and their communities [2,3]. Even more worrisome is that such measures have disproportionately affected disadvantaged groups such as poor people, migrants, internally displaced people, and refugees [2]. These groups of people often live in overcrowded and under-resourced settings and mostly depend on daily labor for subsistence [2]. As a result, most African countries have already realized that lockdowns cannot and should not be enforced indefinitely, as it has already caused enormous damage to economies, livelihoods, health, cultures, behaviors, and is compromising peoples' goodwill and emotional well-being [2,3]. A major concern, therefore, is how African authorities can suppress the spread of SARS-CoV-2 (the virus that causes COVID-19) while at the same time not destroy the economy or undermine peoples' resilience and willingness to consent to strenuous physical and social-distancing practices [2,3].

Like in the United States of America and some countries in Western Europe (places which are still raging full-on with the epidemic) [1], in Africa, the pressing concern now is how to suppress the spread of SARS-CoV-2 to avoid overwhelming the health care systems to provide care for the sick [2-4]. One can reasonably argue that the first objective of any response to COVID-19 is to protect people's lives, which means averting the health care system's collapse to provide care for the sick [1-4]. Hospitals are expected to be the last line of defense in the control and prevention strategy of COVID-19 since they save lives. Given the generally underdeveloped healthcare systems across Africa, we suggest that African governments should implement effective prevention measures for COVID-19 not to get disproportionately high numbers of cases to overwhelm the limited capacity of providing care to all those who need it. In this article, we propose a framework that might guide African governments on how to ease COVID-19 related restrictions and provide specific recommendations on measures that might help limit community transmission of SARS-CoV-2.

A modeling study has demonstrated that the COVID-19 pandemic can substantially be prevented from resurging only when a half or more of the world's population becomes immune to the new virus [5]. Although empirical evidence is still lacking at this point for one to say with certainty, immunity to COVID-19 is expected to be acquired in one of the two ways. The first way is when enough people have been infected and have recovered from the infection (obtained herd immunity). The second way is when groups of people have been inoculated with a vaccine and developed the required antibodies to protect against the virus [5]. Option one of allowing the infection to run through the population uninterrupted could lead to large numbers of deaths, mainly among the most vulnerable populations - the elderly, the poor people with limited access to health care, and individuals with comorbidities such as AIDS, hypertension, diabetes, obesity, and malnutrition among other conditions all of which are prevalent in Africa) [2,5]. Option two of keeping the population safe through physical and social distancing interventions while waiting for a safe and effective vaccine, and making it available for everyone would have been preferable under ideal conditions [2]. Luckily, the rate at which viable vaccines against COVID-19 continue to be developed and rolled-out, especially in the developed world, is unprecedented, thanks to technological advances.

Reports from the USA, for example, have shown a tremendous decline in the number of new cases and hospitalization ever since vaccines were rolled out in January 2021, a sign perhaps that these vaccines are safe and effective against the virus. Even though there are global efforts to make these vaccines globally available via WHO COVAX facility, many developing countries, including many African countries, have not had a fair share of vaccines available for their citizens. So far, very few African countries have initiated the COVID-19 vaccination program. There are already talks of apartheid in vaccine distribution across nations, with some developed nations reportedly reserving vaccine doses beyond what they need for their populations. Although option two (lockdown) has shown promising results in keeping the infection rates low [2,3], we argue that African governments cannot sustainably maintain complete physical and social distancing measures for their populations. Besides the impracticability and the negative social and economic consequences of long-term lockdown measures in most African countries, there is lack of evidence of acquired community-wide herd immunity to SARS-CoV-2, even where it has already hit hardest [5]. Also, there is inadequate information on how many people are known to have been infected and have recovered so far [5]. Due to lack of information on acquired immunity, we recommend serological studies to determine how many people in Africa who have already recovered from infection have sufficient antibodies against SARS-CoV-2.

On charting the way forward, we propose that African governments should consider developing clear, cost plans for lockdown-relax cycles to keep the epidemic under control in their jurisdictions until the recently authorized vaccines become widely available to their populations. Epidemiological data on who is infected, when they are infected, and where they live or work should form the basis for deciding when to lockdown and when to relax. We postulate that the lockdown-relax cycles will keep the COVID-19 pandemic under control yet allow relatively acceptable economic activities to be safely conducted in many communities in Africa. How best to do that will vary from country to country, depending on its means, tolerance for disruption, and peoples' collective will power. It is a three-way tussle, in all scenarios, trying to find a balance amongst combating the disease, protecting the economy, and keeping society on an even keel. The framework on how African governments could monitor the state of the COVID-19 pandemic more accurately, using epidemiological data to fine-tune response interventions quickly enough to stay ahead of the pandemic trajectory, is presented next.

**Use of robust epidemiological data:** the policy for preventing and controlling SARS-CoV-2 need not only be determined based on the daily numbers of reported cases; these numbers of COVID-19 cases we regularly see in daily situational reports; this is so because such numbers are unreliable. Instead, what is needed is the virus's real-time effective reproduction number or the actual ability of SARS-CoV-2 to spread at a particular time. Moreover, one need to understand that number correctly in the context it is used. The rate at which a virus is transmitted, SARS-CoV-2 in this case, is referred to as the basic reproduction number (R0) [6]. Precisely, R0 means the average number of secondary cases arising from one infectious COVID-19 case in a susceptible population, that is, a population with no pre-existing immunity to the virus [6]. Suppose the R0 is more than one (R0 > 1) - in that case, it implies that the number of persons infected by one infectious case is likely to increase. If the R0 is less than one (R0 < 1), it implies that the transmission is expected to reduce and eventually may die out gradually [6]. R0 is an essential indicator of an infectious agent's risk of spreading within a population [6]. R0 can be estimated in the population if pre-existing immunity can be accounted for in the calculation and may vary from one African country to another. Even within the same country, R0 may differ from one place to another mainly due to differences in population dynamics such as age structure and how frequently people come in contact with each other.

The most effective version of R0 is, however, the reproduction number at time “t” (Rt), which is described as the number of people infected by each infected person at time t while public health interventions are in place [5,6]. In other words, Rt is the actual transmission rate of the virus at a given moment in a particular population in the presence of public health interventions [5,6]. Rt varies according to the epidemic control measures that have been put in place. Such epidemic control measures may include; vaccination, herd immunity, quarantine and isolation protocols, travel restrictions, school closures, physical distancing, and use of face masks. In the absence of interventions, it is R0, or in the presence of interventions, the Rt conveys the real state of the SARS-CoV-2 spread rather than the daily reported number of COVID-19 cases. Due to marked differences in disease determinants, each country in Africa and where possible each region within a country should compute their country-specific R0 and Rt to avoid challenges associated with drawing area-specific conclusions about the transmissibility of SARS-CoV-2 based on disparate data.

Another problem associated with making decisions based on the number of reported cases is related to the lag in the actual number of infections by at least 10 to 14 days, the maximum incubation period for SARS-CoV-2 [6]. Moreover, the severe shortages of COVID-19 testing kits in many African Countries result in many people not getting tested timely. Those who get tested probably do so some days after exhibiting symptoms and signs of the disease [2]. If not corrected, all these delays result in a downward bias in the number of new cases by the time of symptom onset towards the day of reporting. Nevertheless, thanks to the open-source software, using both statistical adjustments and digital analytic models, it is now possible for each country to calculate its near-to-actual real-time Rt from the daily reported number of cases. These established statistical methods can provide real-time assessment of the current situation while correcting for under-reporting, in a result called now casting [7].

We suggest that each African country or community determines the Rt it can accept, given its circumstances, notably its health system's capacity to provide care to all those who need it vis-a-vis the stage of the epidemic. As explained earlier, if the Rt equals 1, it means the epidemic is holding steady. That is, for every person infected, another person gets infected, and that another person replaces every one person who either recovers or dies, thus keeping the pool of infected persons at any one time almost the same. If the Rt is less than one, the epidemic will reduce gradually and may eventually die away. Likewise, the epidemic will grow and perhaps exponentially if the Rt is more than 1. Even though a Rt of less than one is preferred for the epidemic to wane away, depending on the context, a Rt of 1 might be acceptable in places with small population size having a few new infections confirmed every day. That said, Rt of 1 would be unacceptable if the epidemic is raging with several hundred or thousands of new cases occurring daily. In the presence of an explosive outbreak, authorities would need to consider first to knock down the Rt to a very low level of, say 0.1 or 0.2, and maintain it for as long as necessary to bring the daily case count down to a manageable level. A modeling study on interventions to reduce COVID-19 mortality and healthcare demand found that a combination of population-wide social distancing, case isolation, quarantine of contacts, and schools and universities' closure can reduce Rt to close to 1 or below, levels required to reduce case incidence rapidly [5]. Such interventions will come in handy for any African government to reduce COVID-19 cases to a manageable level.

**The use of mobile phone data to monitor the population´s compliance to COVID-19 control measures:** African governments and public health responders to the COVID-19 pandemic can and should ethically use data collected by private entities such as telecommunication companies to chart real-time maps that can be used to evaluate compliance to, for example, physical and social distancing interventions [8]. These data mined from mobile phones could be used to determine how people mix, which could infer the likelihood of passing the SARS-CoV-2 around [7,8]. Such data can provide near real-time information about changes in human movement patterns and help refine the interventions [7,8]. With a bit of ingenuity, we believe that existing digital tools in Africa can quickly be turned into COVID-19 monitoring instruments without intruding into peoples' lives. With the continuously increasing access to cellular phones in Africa [7,8], cellular phone activity could provide data on peoples' locations and map their movements in big towns and trading centers in real-time [9,10]. Those who, and rightly so, worry about invading people's privacy should worry less since only aggregate data and, therefore, anonymous numbers rather than personal information or identity will be used in such studies. Equipped with all these tools, public health officials and decision-makers could more precisely adjust response interventions to keep the Rt number at what is acceptable to them for controlling the COVID-19 pandemic within their jurisdiction [9,10].

**Authorities need to determine the number of moderate and severe COVID-19 cases their national health system can effectively handle:** for example, a country with a 32 bed-intensive care unit (ICU) capacity cannot have more than 32 people on respirators at a time. With an average length of a patient's stay in the ICU of 14 days, this country cannot provide intensive care for more than two new patients a day (32/14=2.285). Assuming that about 5% of all newly infected cases are severe to require intensive care, the country cannot afford to have more than a total of 40 new infections a day (2x20=40). Therefore, establishing the number of new COVID-19 cases the country's emergency health facilities can effectively manage helps decision-makers determine the Rt level public health interventions should aim to achieve. Isolation or enhanced shielding of vulnerable populations such as the elderly and those with comorbidities who are likely to get severe forms of COVID is one proposed strategy that may prevent African countries from overwhelming their ICU capacities. As countries ease the lockdown measures, they will quickly need to test suspected cases to identify them quickly, isolate them, find their contacts, and quarantine those contacts if the pools of infection and sources of the infection within the community are to be identified and contained. The country´s health systems' capacity to test and isolate cases is also vital to effectively prevent infection transmission.

**Determine the magnitude of physical and social distancing restrictions that will not destroy the economy and which the citizens are willing to accept:** after determining the number of COVID-19 cases the health care system can effectively handle, decision-makers can determine the magnitude of physical and social distancing restrictions that will not destroy the economy and which the citizens are willing to accept. When choosing which physical and social distancing restrictions to implement at any particular time, it is essential to consider if the economy will not suffocate even if the health care system can tolerate the number of new infections in a given day. Likewise, it is critical to assess how long the population will accept restrictions needed to achieve and maintain the required level of infections. The effects of restrictions on the mental and emotional states of the people should also be assessed. There is anecdotal information that lockdown measures in some African countries have resulted in increased rates of gender-based violence, with some leading to the death of whole families. Due to these dire consequences of restrictions, we strongly recommend African governments to engage the general public, who should help shape the interventions and determine how such interventions should be implemented.

At this point, we wish to point out that there is no right or wrong answer as to what is the best option to respond to a threat as significant and as complex as the COVID-19 pandemic. One can only imagine the variety of personal views. Some people would instead take a chance with COVID-19 rather than jeopardize the economy. In contrast, other people would take no chance with compromising health for fear of economic depression, arguing that economies always bounce back after a year or so, but a lost life is gone forever. We argue that even though different African countries and communities within each country will strike different balances amongst all the factors discussed above, the lockdown-relax strategy's basic tenets apply to all countries and communities. After applying stringent physical and social distancing (total lockdown), which results in a sustained decline in the Rt, and the daily number of new cases is brought down to an acceptable baseline level; authorities can consider relaxing some measures (for example, reopen schools and markets). However, authorities must be ready to re-impose drastic restrictions as soon as the Rt starts rising again above the set target, and the cycle goes on. That means that restrictions must be re-applied and lifted and re-applied and lifted, as long as it takes for the population to build up enough immunity to the virus.

**The lockdown-relaxing cycles:** the importance of lockdown-relaxing cycles is explained further with an example of a worst-case scenario where neither physical distancing nor any other intervention to deter the spread of SARS-CoV-2 is implemented. In such a scenario, R0 will be more than 1, and the virus can infect many people within a few months. As already mentioned, this scenario is unacceptable as it would overwhelm hospitals and lead to higher death rates. The best-case scenario is when Africa's current infection levels are maintained or even better reduced until a vaccine becomes readily available for the whole population. It, therefore, requires a concerted effort on the part of national governments and the population to achieve low infection levels. A considerable degree of effort is needed to implement continued physical distancing, hand washing, and wearing of face masks for extended periods until when the recently authorized, safe and effective vaccines are made available to everyone. Until then, the most likely case scenario is somewhere in the middle, where infection rates increase and decrease over time; authorities may relax physical and social distancing measures when numbers of infections fall and may need to re-implement these measures as numbers increase again. So, a prolonged effort will be required to prevent significant outbreaks. African governments should consider implementing these lockdown - relaxing cycles to control the spread of SARS-CoV-2 while making life manageable for people as vaccines have just come on board. Also, the timing of lockdown and other interventions to suppress the epidemic are crucial for staying within the health system's capacity and minimizing overall mortality.

Our proposed approach of tightening and relaxing measures based on the real-time Rt is similar to the “adaptive triggering” proposed by researchers at Imperial College London, with suppression measures switched on and off depending on whether cases rise or fall [5]. After easing lockdowns, continuing restrictions at some levels are recommended to limit community spread, particularly in hotspots with indications of community transmission. Besides, the mandatory use of face masks in all public areas should be enforced and should go along with regular hand washing with soap and water, use of hand sanitizers, social and physical distancing, community education, sensitization, and engagement on the control and prevention of the spread of COVID-19 and other public health measures already put in place. Other specific indicative recommendations are provided in (Table 1) for high-risk and moderate places or activities. Furthermore, without effective SARS-CoV-2 infection transmission prevention interventions, the number of cases will continue to rise with the potential to overwhelm the system resources to accommodate the growing number of new cases and contacts. With the increasing number of COVID-19 cases, essential health care services would be severely impacted, resulting in increased morbidity and mortality from non-COVID-19 causes [2-4].

**Table 1:** COVID-19 transmission mitigation measures by places, activities and level of risks

Place/activity	Level of risk	Mitigation measures
Markets	High risk	1. Market vendors to attend on alternate schedules
		2. Demarcate and designate specific spaces for each vendor ensuring physical distancing
		3. Mandatory wearing of face masks while in the market for both vendors and their customers
		4. Placement of hand washing stations at appropriate locations and at all market entry and exit gates
		5. Deployment of security to oversee implementation
Public transport	High risk	1.There should be a maximum of half the capacity of passengers in taxis, mini buses, and buses
		2.There should be two passengers in tricycles- keke
		3.There should be one passenger (pilon) on a motorbike (OKADA, Boda-boda)
		4.All occupants including the driver should use face masks
Hospitals	High risk	1.Should institute triage stations at all hospital gates
		2.Place hand washing stations and sanitizers at all hospital gates
		3.Mandatory wearing of face masks for patients and N95 masks for healthcare providers
		4.Social distancing, at least one meter apart
Public gatherings such as funerals	High risk	1.Prohibited for more than 10 people
		2.Place hand washing stations at appropriate locations
		3.Mandatory wearing of face masks for all attendees
		4.Social distancing, at least one meter apart
Public buildings such as Offices and banking halls	Moderate risk	1. Alternate work schedules for the tellers and bankers
		2. Social distancing, at least one meter apart
		3. Mandatory use of face masks for all workers in places where they are in close contact with others
		4. Mandatory use of face mask for anyone who enters the building
		5. Fix a transparent plastic shield between tellers and customers
		6. Disinfection of surfaces including knobs frequently
		7. Place hand washing stations at appropriate locations
		8. Encourage telework

Okada and Boda-boda are commercial motorcycle taxis used widely in Africa in the transport sector

**Proposed preconditions to enhance the effectiveness of the lockdown-relaxing cycle strategy:** we suggest four preconditions that African governments should consider before the reopening process is accomplished if the lockdown-relaxing cycle strategy is to keep the number of new COVID-19infectionslow. There should be: a) Evident decline in the Rt for at least 14 days. b) Sufficient rapid diagnostic testing capacity to test; at minimum, all people with COVID-19 symptoms; including mild cases, close contacts, and those in essential roles. c) An adequately facilitated healthcare system that can safely care for all patients and provide appropriate personal protective equipment for healthcare workers. d) Sufficient public health capacity to conduct contact-tracing for all new cases. We recommend; i) a gradual lifting of lockdown restrictions accompanied by the institution of compulsory preventive measures, such as requirements to wear face masks in public, ensure hand hygiene, and observe physical and social distancing. These measures reflect a “new normal” that may remain long after lockdown measures are lifted; ii) explicitly delaying the reopening of economic activities involving significant physical interactions, such as in schools, places of worship, sporting and exercise facilities, and bars, as has been done in Uganda and Rwanda. In each of those two countries, where businesses have reopened, they are initially subject to the constraint that they operate at reduced capacity to limit physical interaction; iii) a gradual opening up specific regions and businesses and restricting lockdowns to certain hours (instituting curfews at specific hours); iv) enhanced shielding of high-risk persons such as the elderly and persons living with comorbidities; v) completed vaccinations of the most at-risk-populations including health workers. We propose that these recommendations be combined with adaptive triggering to re-impose restrictions (the lockdown-relaxing strategy) if COVID-19 cases begin to rise rapidly. Since some modeling studies suggest that even with a gradual relaxing of some physical distancing measures, we will see infections quickly spread again, we recommend that countries apply a combination of several strategies, such as vaccination, testing, and contact tracing, to further limit community transmission of COVID-19 in communities, after easing the lockdown.

## Conclusion

African governments will need to make and implement explicit rationale evidence-based plans for easing up of restrictions if the low SARS-CoV-2 transmission gains achieved from lockdowns are not to be lost. The post lockdown plans should be based on, for example, local transmission dynamics to determine when and how to open up and when to lockdown again should another epidemic wave happen. Due to the lack of information on the impact of relaxing restrictions of peoples' movement on the transmission of SARS-CoV-2 and how businesses will respond, it is almost clear that there is no single grand lockdown exit strategy. African governments should develop flexible, iterative lockdown exit plans, moving forward while collecting information on the disease epidemiology and on the economic indicators as well as peoples' views at home and from other countries to inform decisions, all of which are paramount for success. A multi-sectoral approach where governments work with multiple stakeholders and sectors, including communities, to design and implement changes and willingness to adapt approaches over time will enable governments to respond according to new evidence. Our proposed framework (lockdown-relax) for easing lockdown is just the kind of technical public health measure that needs to be considered.
